# Response of Rambouillet Lambs to an Artificial Gastrointestinal Nematode Infection

**DOI:** 10.3390/ani12091199

**Published:** 2022-05-06

**Authors:** Jacob W. Thorne, Scott A. Bowdridge, Brenda M. Murdoch, R. Reid Redden

**Affiliations:** 1 Department of Animal, Veterinary and Food Sciences, University of Idaho, Moscow, ID 83844, USA; thor1897@vandals.uidaho.edu (J.W.T.); bmurdoch@uidaho.edu (B.M.M.); 2 Texas A&M AgriLife Research and Extension, San Angelo, TX 76903, USA; 3 Department of Animal and Nutritional Sciences, West Virginia University, Morgantown, WV 26506, USA; scott.bowdridge@mail.wvu.edu

**Keywords:** sheep, genetics, parasites, estimated breeding values

## Abstract

**Simple Summary:**

Meat, milk, and fiber produced from sheep are vitally important resources to society around the world. One of the major challenges to sheep health and performance are parasites, specifically gastrointestinal nematodes. This study was designed to identify the variable responses of a popular breed in the United States, Rambouillet, when inoculated with the gastrointestinal nematode *Haemonchus contortus*. Identification of Rambouillet sheep that have greater natural resistance to *H. contortus* is the first step in breeding sheep that can withstand this parasite. Genetic selection for this trait is critical because *H. contortus* is an extremely prolific parasite that can build up anthelmintic resistance, which leads to reduced efficacy of dewormers. Our results indicate that fecal egg counts, the primary tool for estimating worm population inside the sheep, can have wide variation amongst Rambouillet lambs that were artificially challenged with a known amount of *H. contortus*. We also provide evidence that this difference in fecal egg count is reflective of their sire’s estimated breeding value for this trait. While we describe the response of Rambouillet lambs to *H. contortus*, more research is needed to fully elucidate the mechanism behind resistance and susceptibility of Rambouillet sheep to gastrointestinal nematodes.

**Abstract:**

Gastrointestinal nematodes (GIN) threaten the productivity and health of sheep worldwide, prompting the need for genetic selection to reduce GIN susceptibility. Fecal egg count (FEC), packed-cell volume (PCV), and various production traits were examined in parasitized Rambouillet sheep and compared to sire FEC estimated breeding value (EBV). Rambouillet lambs (*n* = 77) were inoculated with 10,000 *H. contortus* L3 larvae. Subsequently, FEC, PCV, and body weight (BW) were captured at seven-day intervals for six weeks. Lambs were sired by one of two rams with post-weaning FEC EBV of −9% or +9%. Mean FEC differed (*p* = 0.0132) with lambs from the lower EBV sire (“Sire L”) being reduced, versus those from the higher EBV sire (“Sire H”), being 2135 ± 211 vs. 2912 ± 207 eggs per gram, respectively. Males and females did not differ for FEC, but females exhibited a higher mean PCV than males, (33.74 vs. 29.65%, *p* < 0.0001). Lambs were shorn ~120 d post artificial infection and wool measurements were captured. A negative correlation between FEC and grease fleece weight was observed. Our results describe the response of Rambouillet lambs to artificial *H. contortus* infection and suggest FEC EBV can reduce susceptibility to GIN in this breed.

## 1. Introduction

Gastrointestinal nematodes (GIN) are a significant threat to sheep health and productivity in the United States (US) and across the world. Infections with *Haemonchus contortus* (Barber’s Pole Worm), a highly prolific GIN capable of producing 10,000 eggs per day [1], are extremely harmful and potentially fatal to sheep. Furthering the issue, resistance of GIN to all classes of anthelmintics used for treatment of *H. contortus* infection has been observed in the US [2,3]. Sustainable parasite management practices are necessary for controlling GIN in small ruminants, including genetic selection for sheep with greater resistance to *H. contortus* [4].

Rambouillet sheep are integral to the US sheep industry and are noted for producing high quality meat and wool, but are generally considered parasite susceptible. Previous studies have demonstrated that composite Dorset × Rambouillet × Finnish Landrace wool lambs are more susceptible to *H. contortus* than St. Croix, Katahdin, and Barbados Blackbelly × Virgin Island White lambs [5,6,7]. In Merino, the breed from which Rambouillet descended, *H. contortus* infections have been shown to reduce growth performance and wool production [8]. Selection for decreased susceptibility of Merino to *H. contortus* has been on-going for over three decades with lambs from a resistance-selected line performing markedly better under both artificial and natural parasite challenges [9,10,11].

Heritability estimates of post weaning fecal egg counts (FEC) have been reported as 0.15–0.30 in wool lambs [12,13,14,15]. Recently, Rambouillet breeders in the US have begun to submit FEC to the National Sheep Improvement Program (NSIP) to generate estimated breeding values (EBV) for post-weaning FEC, however limited directional selection has occurred to this point. Texas A&M AgriLife Research in San Angelo, TX has been collecting fecal egg counts on Rambouillet lambs and participating in NSIP since 2015, recognizing that an opportunity exists to better elucidate the current susceptibility of this breed as a whole to GIN.

Growth and wool quality have been targeted areas of sheep improvement through management and genetic selection for over a century [16]. Artificially challenging lambs with *H. contortus* can provide valuable baseline data regarding the impact of parasite infection on these key Rambouillet production traits and serve as a foundation for future directional selection for this breed. In composite wool breeds, yearling and mature ewe weight gain was not impeded by a parasite challenge, however the potential impact on fleece weight and quality were not reported [17]. In hair lambs, negative correlations have been observed between post-weaning FEC and post-weaning body weight (BW) [18]. Describing the effect of GIN infection on important traits in Rambouillet and reporting within-breed variation sets a foundation for future selection and management strategies for reducing parasitism and improving animal health and productivity.

Our objective was to measure FEC and PCV to describe the response of Rambouillet lambs challenged artificially with *H. contortus* and to determine how levels of resulting infection impacted their health and production traits, including weight gain and wool growth. We hypothesized that the response of lambs to a controlled challenge of *H. contortus* would result in varied fecal egg counts and that Sire FEC EBV would be predictive of these measurements. We also hypothesized that FEC and PCV would have a negative correlation with production traits.

## 2. Materials and Methods

### 2.1. Lamb Background

Purebred Rambouillet lambs for this study were produced by the Texas A&M AgriLife Research registered Rambouillet flock in San Angelo, Texas (semi-arid climate). In the fall of 2019, ewes from this flock were group-mated to two Rambouillet rams, sire L with a low (−9.9%) or sire H with a high (+9.4) post-weaning FEC EBV. Neither ram had sired offspring prior to this study, and thus the accuracy of these EBVs were low, 44 and 45% for sires L and H, respectively. The Texas A&M AgriLife Research flock had submitted 161 FEC cumulatively over four years to NSIP prior to this study, from which the EBV for sires L and H were developed.

Lambs for this study were born in March of 2020 and were managed with their dams as one cohort on cereal grain fields and native pasture throughout lactation. Parentage was confirmed via DNA analysis with the Flock54 genomic panel (Superior Farms, Sacramento, CA, USA). Lambs were weaned at an approximate age of 60 d. At weaning, lambs were weighed (average = 21.9 kg) and a fecal sample was collected from each lamb and analyzed for strongyle eggs per gram (epg). Fecal egg counts ranged from 0 to 2800 epg, with 92% of the lambs having a FEC > 0 epg. No GIN species identification was conducted, but due to warm environmental conditions and previous coproculture analysis performed on sheep managed at the Texas A&M AgriLife Research station, it was expected that a high percentage of the eggs identified in lamb fecal samples were *H. contortus*. It was determined that all lambs had sufficient exposure to *H. contortus* pre-weaning while on pasture for this trial to be a secondary challenge. Lambs were orally drenched with a full dosage, according to label directions, of Cydectin (0.2 mg/kg; Bayer Animal Health, Shawnee Mission, KS, USA), Valbazen (7.5 mg/kg; Zoetis Inc., Kalamazoo, MI, USA) and Prohibit (8 mg/kg; Agri Laboratories Ltd., St. Joseph, MO, USA) and placed in a dry lot. A follow-up FEC on 20 lambs was conducted two weeks later and no eggs were identified in any of the samples.

Lambs were maintained in two adjacent dry lot pens (30 m × 30 m), divided by sex, consuming a growing ration (12.6% protein) ad libitum for 68 d prior to the initiation of the study. All procedures were approved by the Texas A&M Agriculture Animal Care and Use Committee with Animal Use Protocol #2020-19A.

### 2.2. Lamb Selection

In total, 77 lambs were used in the final analysis of this study, with offspring from both sire L (*n* = 27) and H (*n* = 50) represented. Any lambs that appeared unhealthy, injured, or had a PCV < 20% at any point prior to or during the trial would have been treated accordingly and removed from the study. One lamb did present a PCV of 18% the final week of the study, however, at this time, all lambs were dewormed. Initially, 79 lambs started the trial, but two lambs were removed mid-study for health concerns likely unrelated to the parasite challenge, and their data were removed from the final analysis. Four other lambs were produced from this flock in 2020, however parentage could not be confirmed to be from either Sire L or Sire H, thus they were not included in the study. The 79 lambs that started the trial were selected from 83 total lambs produced from this flock in 2020.

### 2.3. Larvae Recovery

Larvae were sourced from West Virginia University, where they were collected from two Suffolk wethers and developed to the L3 stage via the Baermann technique [19]. The source population of these larvae was confirmed to be >99% *H. contortus* and maintained in isolation to ensure their status as completely susceptible to anthelmintics.

### 2.4. Artificial Challenge Procedures

All lambs were orally inoculated with approximately 10,000 *H. contortus* L3 larvae suspended in 10 mL phosphate-buffered saline (PBS) with a pH of 7.4 at d 0. At this time, lambs were individually weighed, a blood sample was captured in a 16 × 100 mm purple-top blood collection tube containing EDTA from the jugular vein and a fecal sample was collected directly from the rectum. Packed-cell volume (PCV) was determined by filling a 75 mm capillary tube approximately 75% full followed by centrifugation at 4000 rpm for seven minutes with a microhematocrit centrifuge and then subsequently measured for hematocrit percentage.

Fecal samples were placed in an insulated cooler with ice packs immediately after collection and then analyzed the same day. Following a modified McMaster technique protocol, two grams of feces were pulverized and mixed with 28 mL of sodium nitrate solution (specific gravity 1.25–1.30) [20]. After filtering through cheesecloth to remove solids, the solution was placed on a double-sided McMaster slide (Chalex LLC, Wallowa, OR, USA) and strongyle eggs were counted under a microscope using the 10× objective. Eggs per gram was calculated as the total strongyle eggs counted, multiplied by 50.

The collection of FEC, PCV, and body weights was repeated at weekly intervals for six subsequent weeks, with the exception of FEC at week one, as it was expected that developing *H. contortus* were not reproductively mature and producing eggs. To be certain, fecal samples were collected from 20 lambs at week one and no eggs were identified in any of the 20 samples. Average daily gain was calculated as the difference between starting and week six body weights, divided by the duration of days.

Throughout the trial, lambs remained in the same pens as they were assigned to at the initiation of the study and on the same ration, ad libitum. After the final data collection, lambs received a full dose of the anthelmintics Cydectin and Prohibit, according to dosage directions, and follow-up fecal egg counts 14 d later confirmed they were free of parasite eggs.

### 2.5. Wool Data Collection

All lambs were shorn 120 d post artificial infection and wool quality metrics were collected. Grease fleece weight (GFW) was collected at the time of shearing and fiber diameter (FD), fiber diameter standard deviation (FD SD), standard deviation along the fiber (SDA), and staple length (SL) were determined using an OFDA 2000 by staff at the Bill Sims Wool and Mohair Research Laboratory in San Angelo, TX, USA.

### 2.6. Statistical Analysis

Data were analyzed in SAS version 9.4 (SAS Institute Inc., Cary, NC, USA) with generalized linear mixed models (Proc GLIMMIX) with repeated measures. For analysis of FEC, PCV, and average daily gain (ADG), fixed effects included sire, week, sex, and all possible interactions, and initial lamb weight was included as a covariate. When weight was analyzed as the dependent variable, initial weight was not included in the model. For FEC, the model assumed a Poisson distribution to account for non-normality of the data and the ilink option was included in the LSMeans statement to use the natural log transformation of FEC + 100 (to accommodate FEC = 0). Back-transformed least squares means minus 100 for FEC and least squares means for all other traits are reported as these measurements were normally distributed.

Lamb FEC patterns from weeks three through six were also categorized as “low”, “moderate”, “clearing”, or “high”. “Low” lambs had a FEC pattern that did not exceed 4000 epg at any point throughout the trial. “Moderate” lambs recorded a FEC between 4000 and 8000 epg at week six. “Moderate” lambs did not have a decreasing FEC during the final weeks of the trial, thus differentiating them from the “clearing” group, which were lambs that recorded their maximum FEC between 4000 and 8000 epg at either week four or five, but then subsequently decreased in the following collection(s). “High” lambs concluded the trial with a FEC greater than 8000 epg. Proportions of each sire’s offspring in each category were compared using Chi-squared analysis in SAS.

Pearson’s correlation coefficients between FEC, PCV, ADG, and wool quality metrics were calculated using PROC CORR in SAS. Statistical significance was defined as *p* < 0.05 and trends identified for 0.05 < *p* < 0.1.

## 3. Results

### 3.1. Phenotypic Measurements

**Figure 1:**
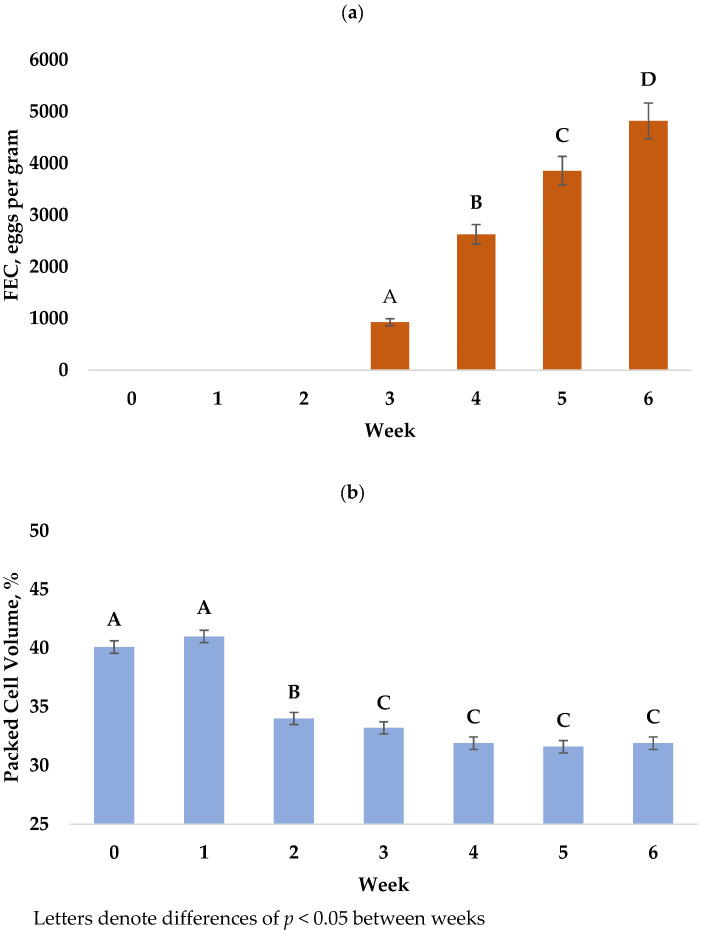
Response of Rambouillet lambs to an inoculation of 10,000 *Haemonchus contortus* L3 larvae. Indicators of parasite infection are displayed and include (**a**) mean fecal egg count and (**b**) mean packed-cell volume percentage for all lambs on the trial.

**Figure 2:**
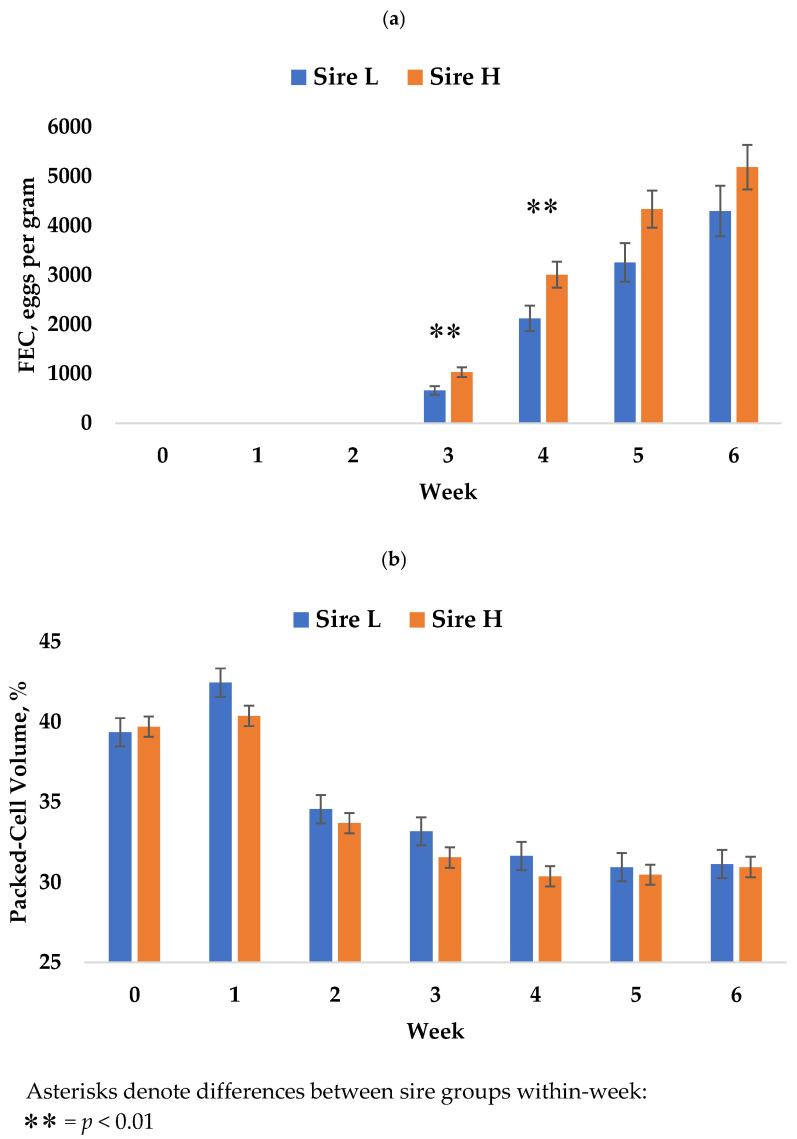
Effects of a *H contortus* infection on Rambouillet lambs. (**a**) Mean fecal egg count (FEC) for all lambs, and for each sire group, for weeks three, four, five, and six. At weeks one and two, FEC were zero. *p*-values for within-week sire comparisons for weeks three, four, five, and six were 0.0082, 0.0094, 0.0503, and 0.2160, respectively. (**b**) Mean packed-cell volume % (PCV) for all lambs, and for each sire group, across the trial.

Measurements of FEC were taken at week two, but lambs did not display FEC above zero until week three, as expected per the reproductive development cycle of *H. contortus*, and thus these data were excluded from the analysis. For all lambs, FEC increased from weeks three through six (*p* < 0.0001) and are reported in Figure 1a. Mean FEC across weeks three through six differed by sire (*p* = 0.0132, Figure 2a), with offspring of sire L (2135 ± 211) having the lower FEC when compared to sire H (2912 ± 207). A sire x week interaction was identified at weeks three and four with FEC of lambs from sire L being lower than lambs from sire H at both time points (*p* = 0.0072, *p* = 0.0222). Lambs from sire L tended to have the lower mean FEC at weeks five (*p* = 0.0555) and no difference was detected at week six (*p* = 0.205). No significant differences in mean FEC between sexes (*p* = 0.6452) or interactions between sex, week, and sire were detected. At week six, male lambs tended to have the lower FEC than females (*p* = 0.0562).

Lamb FEC pattern from weeks three through six was categorized as “low” (*n* = 18), “moderate” (*n* = 35), “clearing” (*n* = 15), or “high” (*n* = 9), with average FECs for these groups displayed in Figure S1. Proportions of each sire’s offspring between groups differed (Figure S2, *p* = 0.0373).

**Figure 3:**
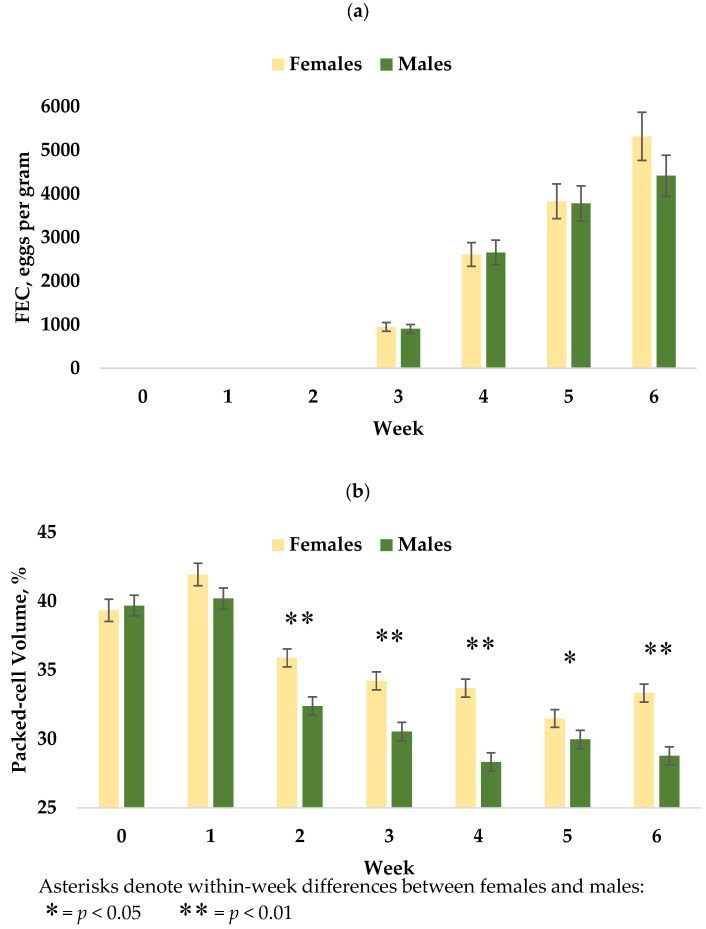
Differing responses to *H. contortus* infection between female and male Rambouillet lambs. (**a**) Fecal egg count by sex. Female lambs tended to have higher FEC at week six (*p* = 0.0562). (**b**) Mean packed-cell volume % (PCV) for lambs by sex. Female lambs appeared to be more “resilient” to H. contortus infection than males, as evidenced by the greater reduction in PCV of male lambs. For all lambs, *H. contortus* larvae had begun feeding on host blood by week two, however FEC were not above zero until week three, confirming that larvae begin consuming host blood prior to sexual maturity.

Packed-cell volume differed by week across the trial (*p* < 0.0001, Figure 1b). Ranges of PCV across trial from the start through week six were 28–56, 27–56, 22–44, 20–42, 20–43, 24–40, and 18–40%, respectively. By sex, PCV also differed (*p* < 0.0001) across the trial and female lambs were significantly higher at week two (36.0 ± 0.7 vs. 32.0 ± 0.7), week three (34.2 ± 0.7 vs. 30.1 ± 0.7), week four (33.7 ± 0.7 vs. 28.1 ± 0.7), week five (31.5 ± 0.7 vs. 29.6 ± 0.7), and week six (33.3 ± 0.7 vs. 28.4 ± 0.7), as displayed in Figure 3. No differences in PCV were observed by sire (Figure 2b) or with all two- and three-way interactions with week and sex.

**Figure 4:**
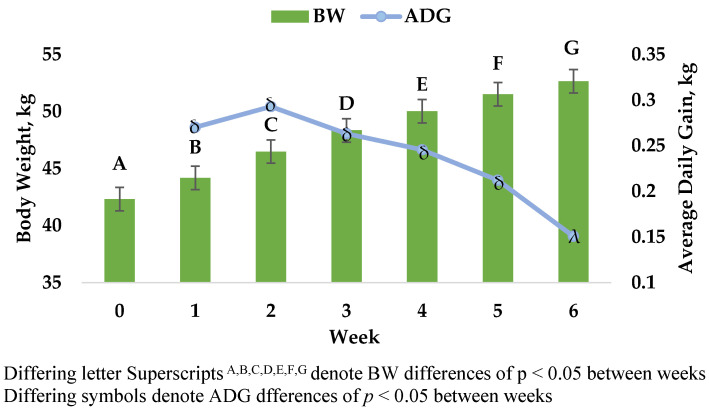
Average lamb body weight (BW), left axis, and average daily gain (ADG), right axis, across the trial of lambs artificially infected with *H. contortus*. Lambs were weighed at weekly intervals and ADG was calculated by subtracting the lamb’s BW at the previous week from the BW of the current week, then dividing by the number of days in between those two measurements.

Differences (*p* < 0.0001) in mean weight at each weekly interval across the trial were observed (Figure 4), with an overall increase in mean weight (including both sexes) from 42.3 to 52.6 kg. Mean ADG did not differ by sire, however males (0.28 ± 0.01 kg/d) gained weight significantly faster than females (0.20 ± 0.01 kg/d; *p* < 0.0001).

### 3.2. Correlations

Wool measurements, collected ~120 d post *H. contortus* infection, are displayed in Table 1. Phenotypic correlations between FEC, PCV, ADG and wool quality metrics are displayed in Table 2. For all lambs, a negative correlation was observed between across-trial mean FEC and GFW and ADG (−0.26, *p* = 0.0294; −0.30, *p* = 0.0101). Average daily gain was positively correlated with GFW, FD, FDSD, SDA, and SL (+0.31, *p* = 0.0088; +0.30, *p* = 0.0092; +0.28, *p* = 0.0176; +0.32, *p* = 0.0069). Interestingly, a negative correlation between ADG and SL was observed (−0.26, *p* = 0.0266).

When correlations were analyzed by sire (Table 3), there was a negative correlation between FEC and both GFW and ADG for sire H (−0.32, *p* = 0.0343; −0.36, *p* = 0.0131). A negative correlation trend was observed between FEC and PCV for sire L (−0.33, *p* = 0.0961). Average daily gain was positively correlated with FD, FDSD, and SDA for sire L (+0.41, *p* = 0.0358; +0.56, *p* = 0.0028; +0.52, *p* = 0.0057). Average daily gain was positively correlated with GFW and FD (+0.34, *p* = 0.0198; +0.37, *p* = 0.01) and tended to be positively correlated with FDSD (+0.27, *p* = 0.0726) for sire H.

When analyzed by sex (Table 4), for female lambs, FEC was negatively correlated with PCV and ADG (−0.42, *p* = 0.0082; −0.44, *p* = 0.007) and positively correlated with SL (+0.34, *p* = 0.034). Neither of these correlations were significant in male lambs, however, in males, FEC was negatively correlated with GFW (−0.35, *p* = 0.0408). Packed-cell volume was also positively correlated with GFW, FD, and ADG in males (+0.41, *p* = 0.0167; +0.37, *p* = 0.0337; +0.37, *p* = 0.0248). Packed-cell volume tended to be positively correlated with ADG in females (+0.30, *p* = 0.0757). In males, ADG was positively correlated with FD, FDSD, and SDA (+0.42, *p* = 0.0131; +0.33, *p* = 0.0483; +0.30, *p* = 0.0764). In females, ADG was negatively correlated with SL (−0.41, *p* = 0.108), and positively correlated with FD (+0.33, *p* = 0.0426), and tended to be positively correlated with GFW and SDA (+0.30, *p* = 0.0654; +0.29, *p* = 0.0741).

## 4. Discussion

### 4.1. Phenotypic Measurements

The mean FEC for all lambs on our study continued to increase from weeks three through six, which is similar to the findings of previous studies with crossbred wool lambs [7]. Though lambs were challenged in a similar method, our FEC results differ from the response exhibited by Katahdin [5], Blackbelly Barbado × Virgin Island White [7], and St. Croix [21], which all peaked at a mean FEC of less than 4000 epg, and then began to show a reduced average FEC by 35 days post-inoculation.

Mean PCV for all lambs in this trial did decrease, as expected, at week 2. Our mean PCV for all lambs is slightly higher than other reports in wool lambs challenged with *H. contortus* [17,22]. However, lambs in our study were being fed ab-libitum and it has been documented that added nutrition can increase PCV, even in the presence of a parasite challenge [23].

Despite the continuous increase of FEC and initial decrease in PCV, lambs continued to gain weight at each weekly interval (Figure 3), also in line with the findings of Notter et al. [7]. Cobon and O’Sullivan [24] reported zero weight gain in Merino lambs that were artificially challenged with 2000 *H. contortus* larvae while being managed on pasture. Average daily gain of lambs in the present study did decrease throughout the trail, but it is unclear if this can be attributed to effects of parasitism or if this is a result of decreasing feed efficiency as lambs reach mature weights, as suggested by Notter et al. [25]. The ADG results in this study are in line with other reports of post-weaning growth in Rambouillet lambs [26].

### 4.2. Response by Sire

Although this study only examined offspring from two rams with divergent EBV, it does support the effectiveness of the post-weaning FEC EBV for reducing parasite susceptibility, as lambs from sire L had a FEC that was 25% lower than those from sire H. This result agrees with Woolaston and Piper [10], who demonstrated that selection of merino sheep over time for FEC EBV increases their resistance.

The FEC EBV of sires L and H were developed from four years of post-weaning FEC collected from lambs on pasture where GIN specie and levels varied. Under typical pasture conditions, parasite species and amount cannot be reasonably controlled for; however, in our study we challenged the lambs with controlled levels of *H. contortus* and the post-weaning FEC EBV of the sires were predictive of their offspring’s averaged response. While Miller et al. [27] described natural challenges as being more useful for identifying genetic variation than artificial challenges in Suffolk and Gulf Coast Native sheep, we have provided evidence that artificial challenge response in Rambouillet can be predicted by data procured in a natural environment. Aguerre et al. [28] also reported a high correlation between FEC from sheep both artificially and naturally challenged.

The greatest differences in FEC by sire were observed at week three (*p* = 0.009) and four (*p* = 0.0222). Although not significant at weeks five and six, it is worth noting that lambs from sire L recorded a lower FEC average at each of these weeks. A possible explanation is that Lambs from Sire L had a more rapid and/or favorable immune response. Escribano et al. [29] reported higher IgA titer levels in wool lambs selected for resistance versus more susceptible contemporaries, with the greatest differences being observed in the two weeks after the lambs were challenged with GIN. Similarly, higher levels of anti-*H. contortus* antibodies were reported by Toscano et al. [30] seven days post-inoculation in resistant versus susceptible Morada Nova lambs. Dargie and Allonby [31] showed that *H. contortus* metabolic activity (and egg output per female worm) were increased in hosts with fewer adult worms, potentially explaining why the FEC of the two sire groups slightly converged in later weeks of the trial. This suggests that lambs from sire L may have more effectively prevented larval establishment. To our knowledge, no other published reports have compared the effectiveness of within-breed FEC EBV as a predictor of offspring performance in a natural and artificial environment.

### 4.3. Fecal Egg Count Patterns

Lamb FEC patterns also suggest potential differences in *H. contortus* establishment and/or reproducing capability (Figure S1). Differences in proportion of offspring were detected (*p* = 0.0373) with 41% of offspring from sire L being classified as having a “low” response as opposed to just 14% for sire H. Further exploration with a greater number of animals is warranted to better understand potential differential responses across a longer time period in Rambouillet following a single inoculation of *H. contortus* larvae.

In Merinos, when a genetically resistant line and a random-bred line were challenged with 20,000 *H. contortus* L3 larvae and then harvested, resistant lambs had approximately half the worm burden of randomly bred lambs [32]. From this same study, peripheral blood mononuclear cells (PBMC) had higher proliferation rates in response to *H. contortus* antigens in resistant lambs versus their randomly bred counter parts. Almost three decades later, sheep from this genetically resistant line were used in a transcriptome analysis by Zhang et al. [33] in which they identified 127 genes displaying a difference in abundance between resistant and susceptible animals. Many of these genes identified are involved in B-cell canonical pathways, agranulocyte and granulocyte adhesion, and diapedesis pathways [33].

### 4.4. Response by Sex

No statistical difference between the sexes in mean FEC was observed, though at week 6, male lambs tended to be lower than females (4341 ± 425 vs. 5710 ± 580 epg, *p* = 0.0562). Interestingly, female lambs had the higher across-trial mean PCV (Figure 3). Higher PCV averages in female lambs than males after an artificial challenge have been reported in hair lambs [34], Dorset x Rambouillet x Finnish Landrace wool lambs [7], and in German wool breeds [35]. In contrast, Woolaston et al. [9] reported higher PCV in male Merino lambs than females under a natural infection but found no sex difference when a cohort from the same source population was challenged artificially.

The differences in hematocrit observed in this study remain unclear and further research is necessary to fully understand these observations. Zeng et al. [36] described polymorphisms in erythropoietin (*EPO*) and its receptor (*EPOR*) that were associated with increased PCV% and were more prevalent in male humans. Our findings suggest that female Rambouillet lambs are not as negatively affected (more resilient) by an *H. contortus* infection as males. It does not appear that sire FEC EBVs are predicative of PCV in Rambouillet lambs, as within-sex sire group analysis did not detect significant differences.

### 4.5. Correlations with Wool Measurements

The value of wool from Rambouillet sheep is increased with heavier weight fleeces (greater volume), fineness of the individual fibers, and the strength of those fibers [37]. Wool quality has been shown to be reduced in Merino sheep for 14 weeks following infection with *H. contortus* [38]. Strong negative correlations between fecal egg count and GFW and CFW have also been observed in South African Dohne sheep [39]. In line with these findings, we identified a negative correlation between FEC and GFW, and a positive correlation between FEC and FDSD. It is important to note that in the present study, shearing occurred 4 months after the lambs were challenged with *H. contortus*, thus the effect of parasites on wool quality cannot be directly determined from these results. Nevertheless, the implications from our results are potentially two-fold: (1) stress from an increased worm burden could potentially decrease wool production and (2) selection for a decreased FEC might be correlated with improve wool growth and fiber strength. Pollot et al. [40] found that genetic correlations between FEC and wool traits are not sensitive to environments regardless of how conducive it is to parasites.

A negative correlation between FEC and both GFW and ADG was observed in the present study, but when separated by sire these two correlations were only still observed in lambs from Sire H, and not Sire L. It is important to consider a potential genetic disparity for wool traits between these two sires that may have impacted their lamb’s measurements. Notably, the yearling FD EBV for sires L and H were −0.99 and + 0.32 microns, respectively, at the beginning of this trial. Mean FD of lambs by sire were 17.59 ± 1.44 and 18.48 ± 1.37 for rams L and H (*p* = 0.0081), respectively. It is possible that differences observed in wool quality by sire are due to genetic factors unrelated to parasite susceptibility.

Female lambs had strong negative correlation between FEC and PCV, an interesting result, as male lambs had the lower overall PCV. Despite this, the positive correlations between PCV and both GFW and FD indicate that *H. contortus* infections in less resilient males potentially had lasting impacts on wool measurements.

### 4.6. Breed Implications

Rambouillet sheep are recognized for their multi-purpose capabilities of producing high quality meat and wool. This study provides information about the response of Rambouillet lambs to an artificial GIN infection protocol, a foundation from which future research in this breed can expand upon. Our findings also provide insight into the predictive capabilities of FEC EBV in this scenario. The nexus between parasites, wool quality, and breed type will need to be further explored, yet correlations observed in this study highlight the negative impact of *H. contortus* infection on Rambouillet fleece characteristics.

## 5. Conclusions

Our results demonstrate the varied response of Rambouillet lambs to an artificial parasite challenge. Additionally, our FEC results by sire were in line with predictions from sire post-weaning FEC EBV, confirming our hypothesis. To fully explore how effective artificial challenges, using species-specific GIN, are for breeding Rambouillet sheep with improved parasite resistance, further studies are required. We have also provided evidence that artificial challenges can be used for identifying variable responses of Rambouillet lambs for aid in future genetic selection strategies. Though more research is needed, genetic selection for parasite resistance will improve the overall health of flocks challenged with GIN and likely reduce the negative impact GIN have on correlated traits such as wool production and lamb growth.

## Figures and Tables

**Table 1:** Wool measurements by sire and by sex of lambs artificially infected with *H. contortus*. Data include grease fleece weight (GFW), fiber diameter (FD), fiber diameter standard deviation (FDSD), standard deviation along the wool fiber (SDA), and staple length (SL).

	GFW (g)	FD (µ)	FD SD (µ)	SDA (µ)	SL (mm)
**Sire H**	2.82 ^A^	18.48 ^A^	3.37 ^A^	1.17 ^A^	54.96 ^A^
**Sire L**	2.73 ^A^	17.59 ^B^	3.15 ^B^	1.09 ^A^	49.77 ^B^
*p*-Value	0.5559	0.0081	0.0379	0.3512	0.0072
**Female**	2.57 ^A^	17.89 ^A^	3.18 ^A^	1.05 ^A^	52.64 ^A^
**Male**	2.98 ^B^	18.18 ^A^	3.34 ^A^	1.21 ^A^	52.97 ^A^
*p*-Value	0.0049	0.3799	0.1296	0.0603	0.7050

Differing letter superscripts ^A,B^ denote between week differences of *p* < 0.05.

**Table 2:** Pearson’s correlation coefficients (with *p*-values located below each correlation) between fecal egg count (FEC), packed-cell volume (PCV), and average daily gain (ADG) and grease-fleece weight (GFW), fiber diameter (FD), standard deviation along the fiber (SDA), fiber diameter standard deviation (FDSD), and staple length (SL) in Rambouillet lambs. Lambs were sheared 4 months after an artificial *H. contortus* challenge, at approximately one year of age. Significant correlations and trends are bolded.

	FEC	PCV	ADG	GFW	FD	SDA	FDSD
**PCV**	−0.14369						
	0.2156						
**ADG**	−0.2974 **	−0.0791					
	**0.0101**	0.4999					
**GFW**	−0.2569 **	0.05323	0.38461 ***				
	**0.0294**	0.6547	**0.0009**				
**FD**	0.02556	0.13227	0.31994 ***	0.38591 ***			
	0.8312	0.2646	**0.0061**	**0.0006**			
**SDA**	0.08128	−0.05086	0.35613 ***	0.33355 ***	0.37471 ***		
	0.4973	0.6691	**0.0021**	**0.0032**	**0.0009**		
**FDSD**	0.16806	−0.07838	0.33943 ***	0.20477 *	0.65691 ***	0.63361 ***	
	0.1582	0.5098	**0.0035**	**0.076**	**<0.0001**	**<0.0001**	
**SL**	0.19473	0.00681	−0.26129 **	0.17444	0.13606	0.22163 *	0.11501
	0.1012	0.9544	**0.0266**	0.1318	0.2412	**0.0543**	0.3225

* *p* < 0.1, ** *p* < 0.05, *** *p* < 0.01.

**Table 3:** Pearson’s correlation coefficients by sire (with *p*-values located below each correlation) between fecal egg count (FEC), packed-cell volume (PCV), and average daily gain (ADG) and grease-fleece weight (GFW), fiber diameter (FD), fiber diameter standard deviation (FDSD), and standard deviation along the fiber (SDA) and staple length (SL) in Rambouillet lambs. Lambs were sheared 4 months after an artificial *H. contortus* challenge, at approximately one year of age. Significant correlations and trends are in bold.

	Sire L			Sire H		
	FEC	PCV	ADG	FEC	PCV	ADG
PCV	−0.32684 *			0.03402		
	**0.0961**			0.8165		
ADG	−0.17118	−0.04901		−0.35928 **	−0.12551	
	0.3933	0.8082		**0.0131**	0.3953	
GFW	−0.21717	0.09607	0.30245	−0.31625 **	0.01902	0.46951 ***
	0.2765	0.6336	0.1252	**0.0343**	0.9002	**0.0011**
FD	0.0661	0.09839	0.40557 **	−0.1173	0.23653	0.37419 **
	0.7432	0.6254	**0.0358**	0.4428	0.1135	**0.0113**
SDA	0.15714	0.01963	0.55009 ***	−0.00923	−0.10817	0.21737
	0.4337	0.9226	**0.003**	0.952	0.4742	0.1515
FDSD	0.29924	−0.27917	0.58192 ***	0.02376	0.10888	0.2702 *
	0.1294	0.1585	**0.0015**	0.8769	0.4714	**0.0726**
SL	0.02513	−0.1687	−0.3065	0.20979	0.18149	−0.19737
	0.901	0.4003	0.12	0.1666	0.2274	0.1938

* *p* < 0.1, ** *p* < 0.05, *** *p* < 0.01.

**Table 4:** Pearson’s correlation coefficients by sex (with *p*-values located below each correlation) between fecal egg count (FEC), packed-cell volume (PCV), and average daily gain (ADG) and grease-fleece weight (GFW), fiber diameter (FD), fiber diameter standard deviation (FDSD), and standard deviation along the fiber (SDA) and staple length (SL) in Rambouillet lambs. Lambs were sheared 4 months after an artificial *H. contortus* challenge, at approximately one year of age. Significant correlations and trends are bolded.

	Females			Males		
	FEC	PCV	ADG	FEC	PCV	ADG
PCV	−0.42268 ***			−0.1069		
	**0.0082**			0.5229		
ADG	−0.43587 ***	0.29157 *		0.08874	0.36843 **	
	**0.007**	**0.0757**		0.6015	**0.0248**	
GFW	−0.09589	0.12254	0.30195 *	−0.3527 **	0.40776 **	0.1506
	0.5668	0.4574	**0.0654**	**0.0408**	**0.0167**	0.3952
FD	0.00561	0.03155	0.33062 **	0.12039	0.3652 **	0.45686 ***
	0.9733	0.8488	**0.0426**	0.4976	**0.0337**	**0.0066**
FDSD	0.2067	−0.06089	0.21269	0.04595	0.20845	0.36846 **
	0.2131	0.7127	0.1998	0.7964	0.2368	**0.032**
SDA	0.20991	−0.13877	0.29307 *	0.19956	0.15185	0.49845 ***
	0.2059	0.3995	**0.0741**	0.2578	0.3913	**0.0027**
SL	0.34478 **	−0.11175	−0.41932 ***	−0.14048	0.06443	−0.17065
	**0.034**	0.4982	**0.0088**	0.4281	0.7173	0.3346

* *p* < 0.1, ** *p* < 0.05, *** *p* < 0.01.

## Supplementary Materials

The following supporting information can be downloaded at: https://www.mdpi.com/article/10.3390/ani12091199/s1, Figure S1: Lamb fecal egg count patterns, Figure S2: Proportion of sire’s offspring with different fecal egg count patterns.

## References

[1] Roberts J.L., Swan R.A. (1981). Quantitative studies of ovine haemonchosis. I. Relationship between faecal egg counts and total worm counts. Vet. Parasitol..

[2] Kaplan R.M. (2004). Drug resistance in nematodes of veterinary importance: A status report. Trends Parasitol..

[3] Howell S.B., Burke J.M., Miller J.E., Terrill T.H., Valencia E., Williams M.J., Williamson L.H., Zajac A.M., Kaplan R.M. (2008). Anthelmintic resistance on sheep and goat farms in the southeastern United States. JAVMA.

[4] Burke J.M., Miller J.E. (2020). Sustainable approaches to parasite control in ruminant livestock. Vet. Clin. N. Am. Food Anim. Prac..

[5] Vanimisetti H.B., Greiner S.P., Zajac A.M., Notter D.R. (2004). Performance of hair sheep composite breeds: Resistance of lambs to *Haemonchus contortus*. J. Anim. Sci..

[6] Zajac A.M., Krakowka S., Herd R.P., McClure K.E. (1990). Experimental *Haemonchus contortus* infection in three breeds of sheep. Vet. Parasitol..

[7] Notter D.R., Andrew S.A., Zajac A.M. (2003). Responses of hair and wool sheep to a single fixed dose of infective larvae of *Haemonchus contortus*. J. Anim. Sci..

[8] Albers G.A.A., Gray G.D., Le Jambre L.F., Piper L.R., Barger I.A., Barker J.S.F. (1989). The effect of *Haemonchus contortus* on liveweight gain and wool growth in young merino sheep. Aust. J. Agric. Res..

[9] Woolaston R.R., Barger I.A., Piper L.R. (1990). Response to helminth infection of sheep selected for resistance to *Haemonchus contortus*. Int. Nat. J. Parasitol..

[10] Woolaston R.R., Piper L.R. (1996). Selection of Merino sheep for resistance to *Haemonchus contortus*: Genetic variation. Anim. Sci..

[11] Karlsson L.J.E., Greeff J.C. (2006). Selection response in fecal worm egg counts in the Rylington Merino parasite resistant flock. Aust. J. Exp. Agric..

[12] Albers G.A.A., Gray G.D., Piper L.R., Barker J.S.F., Le Jambre L.F., Barger I.A. (1987). The genetics of resistance and resilience to *Haemonchus contortus* infection in young merino sheep. Int. J. Parasitol..

[13] Brown D.J., Fogarty N.M. (2016). Genetic relationships between internal parasite resistance and production traits in Merino sheep. Anim. Prod. Sci..

[14] Li L., Brown D.J., Swan A.A., van der Werf J.H.J. (2018). Genetic parameters for faecal worm egg count at different ages in Australian sheep under natural challenge. Anim. Prod. Sci..

[15] Snyman M.A., Fisher A.D. (2019). Genetic parameters for traits associated with resistance to *Haemonchus contortus* in a South African Dohne Merino sheep flock. Small Rum. Res..

[16] Thorne J.W., Murdoch B.M., Freking B.A., Redden R.R., Murphy T.W., Taylor J.B., Blackburn H.D. (2021). Evolution of the sheep industry and genetic research in the United States: Opportunities for convergence in the twenty-first century. Anim. Gen..

[17] Vanimisetti H.B., Andrew S.L., Zajac A.M., Notter D.R. (2004). Inheritance of fecal egg count and packed cell volume and their relationship with production traits in sheep infected with *Haemonchus contortus*. J. Anim. Sci..

[18] Ngere L., Burke J.M., Morgan J.L.M., Miller J.E., Notter D.R. (2018). Genetic parameters for fecal egg counts and their relationship with body weights in Katahdin lambs. J. Anim. Sci..

[19] Zajac A., Conboy G. (2006). Veterinary Clinical Parasitology.

[20] Whitlock H.V. (1948). Some modifications of the McMaster helminth egg-counting technique and apparatus. J. Counc. Sci. Indust. Res..

[21] Jacobs J.R., Greiner S.P., Bowdridge S.A. (2015). Serum interleukin-4 (IL-4) production is associated with lower fecal egg count in parasite-resistant sheep. Vet. Parsitol..

[22] Amarante A.F.T., Craig T.M., Ramsey W.S., Davis S.K., Bazer F.W. (1999). Nematode burdens and cellular responses in the abomasal mucosa and blood of Florida Native, Rambouillet and crossbreed lambs. Vet. Parasitol..

[23] Datta F.U., Nolan J.V., Rowe J.B., Gray G.D. (1998). Protein supplementation improves the performance of parasitized sheep fed a straw-based diet. Int. J. Parasitol..

[24] Cobon D.H., O’Sullivan B.M. (1992). Effect of *Haemonchus contortus* on productivity of ewes, lambs and weaners in a semi-arid environment. J. Agric. Sci..

[25] Notter D.R., Ferrell C.L., Field R.A. (1984). Effects of breed and intake level on growth and feed efficiency in ram lambs. J. Anim. Sci..

[26] Yeaman J.C., Waldron D.F., Willingham T.D. (2013). Growth and feed conversion efficiency of Dorper and Rambouillet lambs. J. Anim. Sci..

[27] Miller J.E., Bishop S.C., Cockett N.E., McGraw R.A. (2006). Segregation of natural and experimental gastrointestinal nematode infection in F2 progeny of susceptible Suffolk and resistant Gulf Coast Native sheep and its usefulness in assessment of genetic variation. Vet. Parasitol..

[28] Aguerre S., Jacquiet P., Brodier H., Bournazel J.P., Grisez C., Prévot F., Micho L., Fidelle F., Astruc J.M., Moreno C.R. (2018). Resistance to gastrointestinal nematodes in dairy sheep: Genetic variability and relevance of artificial infection of nucleus rams to select for resistant ewes on farms. Vet. Parasitol..

[29] Escribano C., Saravia A., Costa M., Castells D., Ciappesoni G., Riet-Correa F., Freire T. (2019). Resistance to *Haemonchus contortus* in Corriedale sheep is associated to high parasite-specific IgA titer and a systemic Th2 immune response. Sci. Rep..

[30] Toscano J.H.B., Okino C.H., Barbosa dos Santos I., Giraldelo L.A., Borsch von Haehling M., Novita Esteves S., Bassetto C.C., Talamini do Amarante A.F., de Souza Chagas A.C. (2020). Local and systemic immune mediators of Morada Nova lambs with divergent *Haemonchus contortus* resistance phenotypes. Parasite Immunol..

[31] Dargie J.D., Allonby E.W. (1975). Pathophysiology of single and challenge infections of *Haemonchus contortus* in Merino sheep: Studies on red cell kinetics and the “self-cure” phenomenon. Intl. J. Parasitol..

[32] Gill H.S. (1994). Cell-mediated immunity in Merino lambs with genetic resistance to *Haemonchus contortus*. Int. J. Parasitol..

[33] Zhang R., Liu F., Hunt P., Li C., Zhang L., Ingham A., Li R.W. (2019). Transcriptome analysis unraveled potential mechanisms of resistance to *Haemonchus contortus* infection in Merino sheep populations bred for parasite resistance. Vet. Res..

[34] Toscano J.H.B., Dos Santos I.B., von Haehling M.B., Giraldelo L.A., Lopes L.G., da Silva M.H., Figueiredo A., Esteves S.N., de Souza Chagas A.C. (2019). Morada Nova sheep breed: Resistant or resilient to *Haemonchus contortus* infection?. Vet. Parasitol..

[35] Gauly M., Schackert M., Hoffmann B., Erhardt G. (2006). Influence of sex on the resistance of sheep lambs to an experimental *Haemonchus contortus* infection. Dtsch. Tierarztl. Wochenschr..

[36] Zeng S.M., Yankowitz J., Widness J.A., Strauss R.G. (2001). Etiology of differences in hematocrit between males and females: Sequence-based polymorphisms in erythropoietin and its receptor. JGSM.

[37] Holman B.W.B., Malau-Aduli A.E.O. (2012). A review of sheep wool quality traits. Annu. Rev. Res. Biol..

[38] Albers G.A.A., Gray G.D., Le Jambre L.F., Barger I.A., Barker J.S.F. (1990). The effect of *Haemonchus contortus* infection on haematological parameters in young Merino sheep and its significance for productivity. Anim. Sci..

[39] Matebesi-Ranthimo P.A., Cloete S.W., Van Wyk J.B., Olivier J.J. (2014). Genetic parameters and relationships of faecal worm egg count with objectively measured wool traits in the Tygerhoek Merino flock. S. Afri. J. Anim. Sci..

[40] Pollott G.E., Karlsson L.J.E., Eady S., Greeff J.C. (2004). Genetic parameters for indicators of host resistance to parasites from weaning to hogget age in Merino sheep. J. Anim. Sci..

