# Supplementary material for: Response of Rambouillet Lambs to an Artificial Gastrointestinal Nematode Infection
Source: Animals (Basel). 2022 May 6;12(9):1199. doi: 10.3390/ani12091199 (PMC9102365; doi:10.3390/ani12091199)
Supplement: Supplementary file 1 [file animals-12-01199-s001.zip › Supplemental Figure S1.pdf]

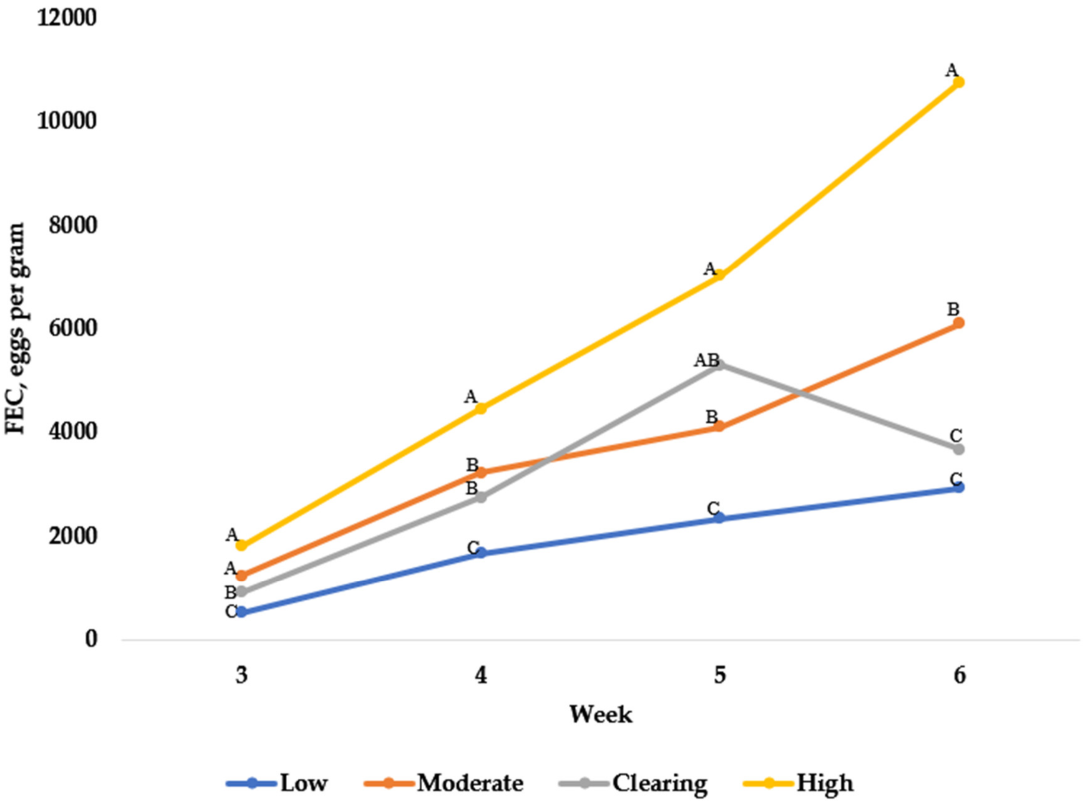

Differing letter Superscripts ^{A,B,C} denote between week differences of $P < 0.05$

Supplemental Figure S1. Fecal egg counts of lambs that completed the trial (N = 77) at weeks three, four, five, and six display an apparent differing response to a single inoculation of *Haemonchus contortus* larvae. "Low" lambs (N = 18) displayed a weekly FEC pattern that remained below 4000 epg for the entirety of the trial. "Moderate" lambs (N = 36) had a FEC between 4000 and 8000 epg at week six and did not display a "clearing" response. "Clearing" lambs (N = 12) had a peak FEC at weeks four or five and then subsequently decreased. "High" lambs (N = 11) displayed a FEC greater than 8000 epg at week 6. Mean FEC for each group is charted on the figure.
