# Supplementary material for: Response of Rambouillet Lambs to an Artificial Gastrointestinal Nematode Infection
Source: Animals (Basel). 2022 May 6;12(9):1199. doi: 10.3390/ani12091199 (PMC9102365; doi:10.3390/ani12091199)
Supplement: Supplementary file 1 [file animals-12-01199-s001.zip › Supplemental Figure S2.pdf]

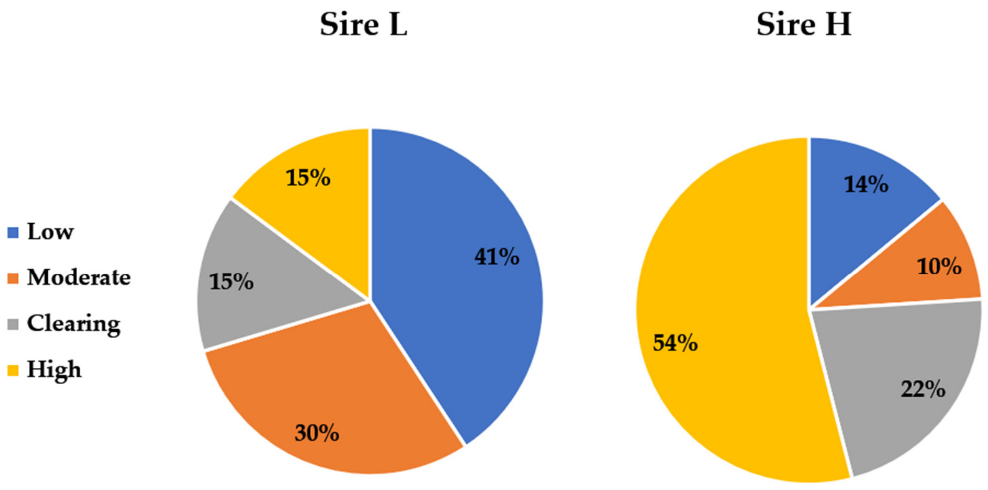

Supplemental Figure S2. Proportion of offspring from sires L (N = 27) and H (N = 50) that displayed either a "low", "moderate", "clearing", or "high" FEC pattern. Proportion of each sire's offspring within these classifications differed from expected values via chi-square analysis ($p = 0.0373$).
